# A Novel Lipase as Aquafeed Additive for Warm-Water Aquaculture

**DOI:** 10.1371/journal.pone.0132049

**Published:** 2015-07-06

**Authors:** Chao Ran, Suxu He, Yalin Yang, Lu Huang, Zhigang Zhou

**Affiliations:** Key Laboratory for Feed Biotechnology of the Ministry of Agriculture, Feed Research Institute, Chinese Academy of Agricultural Sciences, Beijing, P. R. China; Huazhong university of Science and Technology, CHINA

## Abstract

A novel *Acinetobacter* lipase gene *lipG1*was cloned from DNA extracted from intestinal sample of common carp (*Cyprinus carpio*), and expressed in *E*. *coli* BL21. The encoded protein was 406 amino acids in length. Phylogenetic analysis indicated that LipG1 and its relatives comprised a novel group of true lipases produced by Gram-negative bacteria. LipG1 showed maximal activity at 40℃ and pH 8.0 when *p*NP decanoate (C10) was used as the substrate, and remained high activity between 20℃ and 35℃. Activity of the lipase was promoted by Ca^2+^ and Mg^2+^, and inhibited by Zn^2+^ and Cu^2+^. Moreover, LipG1 is stable with proteases, most commercial detergents and organic solvents. Substrate specificity test indicated that LipG1can hydrolyse *p*NP esters with acyl chain length from C2 to C16, with preference for medium-chain *p*NP esters (C8, C10). Lastly, LipG1was evaluated as an aquafeed additive for juvenile common carp (*Cyprinus carpio*). Results showed that supplementation of LipG1significantly improved the gut and heptaopancreas lipase activity of fish fed with palm oil diet. Consistently, improved feed conversion ratio and growth performance were recorded in the LipG1 feeding group, to levels comparable to the group of fish fed with soybean oil diet. Collectively, LipG1 exhibited good potential as an aquafeed additive enzyme, and deserves further characterization as the representative of a novel group of lipases.

## Introduction

China is the largest aquaculture producer in the world, with a total production of 53.94 million tons of fish in 2012, accounting for nearly 60% of the world total [1]. Consequently, there has been a consistent increase in the demand for aquafeed over time. However, the price of aquafeed is relatively high, which represents one of the main bottlenecks for the development of aquaculture. In aquafeed, dietary lipids provide essential fatty acids and are important source of energy for fish. The optimum dietary lipid levels for tilapia and common carp are as high as 8% and12%, respectively [2]. Fish oil and soybean oil have been used as the lipid source in aquafeed for these fish species. However, due to the high price of both fish oil and soybean oil, as well as the finite nature of fish oil, alternative vegetable oils are prevalent. Among these, palm oil (PO) is the vegetable oil with the highest production volume and lowest price worldwide, and therefore is a very attractive lipid source for reduction of the aquafeed price.

Fatty acids (FA) digestibility in fish decreases with increasing chain length and increases with increasing degree of unsaturation [3, 4, 5]. Also, the digestibility of FA is influenced by dietary lipid source and the level of dietary saturates [6], i.e., high level of saturated fatty acids in the diet may reduce the digestibility of saturated FA, and to a lesser extent, of the monoenes and PUFA [3, 4]. Although research into the use of palm oil in fish diets has shown encouraging results [7–10], supplementation of palm oil in aquafeed may reduce the digestibility of lipid and fatty acids, especially when used at high dietary level and low temperature, due to its high level of saturated fatty acids [3, 4, 8]. The reduction in lipid and fatty acids digestibility could lead to compromised growth and feed utilization efficiency of fish, given a lower level of total lipid in the aquafeed and longer feeding period [4, 8]. The reduction of fatty acids digestibility of high PO diet, especially saturates, was partially due to the poor triacylglycerol (TAG) digestion, as indicated by the increased TAG content in fecal lipids of fish fed high PO diet [3]. Therefore, a potential way to solve the problem is supplementation of exogenous lipase in the diet to improve the lipolysis of TAGs of palm oil.

The studies on the supplementation of exogenous lipase for improvement of lipid digestion and growth performance have rarely been conducted in fish. Samuelsen et al. reported that lipase addition had no effect on growth performance and related parameters of rainbow trout (*Oncorhynchus mykiss*) [11]. Moreover, properties of many available lipases were not suitable as aquafeed additive.

Microbial lipases have been widely utilized due to their availability, substrate specificity, and diversity in catalytic activities [12, 13]. In particular, many novel lipases have been isolated or cloned from *Acinetobacter* spp., showing various beneficial biochemical properties such as stability in organic solvents, broad substrate specificity, cold-adapted ability and stereo-selectivity [14–19]. In this study, a novel *Acinetobacter* lipase gene was cloned from DNA extracted from intestinal sample of common carp, *Cyprinus carpio*, and subsequently expressed in *E*. *coli*. Biochemical properties of the lipase were characterized, and the application of this lipase as aquafeed additive was evaluated in common carp fed diet with high level of palm oil.

## Materials and Methods

### Ethics statement

All experimental and animal care procedures were approved by Feed Research Institute of Chinese Academy of Agricultural Sciences Animal Care Committee, under the auspices of the China Council for Animal Care (Assurance # 2012-HSX01). MS-222 was used as the anaesthetic. Field study was conducted on private aquaculture pond under the owner's permission.

### Strains and media

*E*. *coli* DH5α was used for cloning of the PCR product. *E*. *coli* BL21 was used for expression of the recombinant enzyme. *E*. *coli* strains were cultured in LB medium (10 g tryptone, 5 g yeast extract, 10 g NaCl per liter) at 37°C.

### Cloning of the lipase gene and sequence analysis

By comparing the amino acid sequences of *Acinetobacter* lipases, two highly conserved regions were identified. The degenerate primers were designed based on the sequences of the two regions by CODEHAP primer [20]. The partial *Acinetobacter* lipase gene sequences between the two conserved motifs were amplified from DNA extracted from intestinal sample of common carp by touchdown PCR. The amplified PCR product was cloned into *E*. *coli* DH5α using pGEM-T Easy (Promega, Madison, USA). Fifty transformants were randomly picked. Plasmids were extracted with the TIANprep Mini Plasmid Kit (TIANGEN, Beijing, China), and the cloned partial lipase gene sequences were sequenced at Beijing Calculation Centre. One sequence that showed relatively high abundance among the sequenced clones was selected for further study. To obtain the upstream and downstream of the partial sequence of the lipase gene, genome walking PCR was performed with a genome walking kit according to manufacturer's instructions (Takara, Japan). The primers were designed based on the partial sequence (Table 1). The elongated fragment was sequenced at Beijing Calculation Centre. Sequence assembly was performed using Vector NTI Suite 7.0 (InforMax, Gaithersburg, MD, USA). ORF was found using the alternative initiation codon finder in OR finder (http://www.ncbi.nlm.nih.gov/projects/gorf/). The amino acid sequence was analyzed by SignalP 3.0 server [21] to identify the signal peptide. Alignments of the DNA and protein sequences were carried out using BLASTn and BLASTp, respectively (http://www.ncbi.nlm.nih.gov/BLAST/). Phylogenetic tree was generated using the neighbor-joining method by MEGA 5.0 [22].

**Table 1:** Primers used for gene cloning and expression.

	Sequences	bp
Acinet alkaline F	5'-GGCACATGGCACAACAGGNGTNGSNGA-3'	27
Acinet alkaline R	5'- GAGCTGCATGTCCGCCYTGNSWNTG-3'	25
AG1 usp1	5'- GGTCATCCAGCGATTCGACACTT-3'	23
AG1 usp2	5'- CTGCCACTACGGCGTCAGTAATTG-3'	24
AG1 usp3	5'- TTAATTGACTGCGACTTGGTGCAC-3'	24
AG1 dsp1	5'- TTGTGCACCAAGTCGCAGTCAAT-3'	23
AG1 dsp2	5'- TTAACTGCGGGCTATGTGGTGGG-3'	23
AG1 dsp3	5'- AGTGTCGAATCGCTGGATGACC-3'	22
AG1 dsp2	5'- TTAACTGCGGGCTATGTGGTGGG-3'	23
AG1 dsp3	5'- AGTGTCGAATCGCTGGATGACC-3'	22
lipG1 F	5'-TAGGAATCCCATGATGATGATCATGATTCTTATC-3'	34
lipG1 R	5'-ATTTGCGGCCGCTTGAATTGGCATTAATGTTTGTAC-3'	36
338-GC-f	5’-CGCCCGCCGCGCGCGGCGGGCGGGGCGGGGGCACGGGGGGACTCCTACGGGAGGCAGCAG-3’	60
519 r	5'-ATTACCGCGGCTGCTGG-3'	17

### Expression of the recombinant lipase in *E*. *coli*

The following primers were used for construction of the lipase expression plasmid: lipG1F (with *Eco*RI site) and lipG1 R (with *Not*I site). The PCR products were inserted into pET-28a (+) digested with *Eco*RI and *Not* I. The recombinant plasmid, pET28a—lipG1, was sequenced to confirm the insertion, followed by transformation into *E*. *coli* BL21 (DE3) cells. Transformed cells were picked from a single colony and grown overnight at 37°C in shake flask containing 3 ml LB broth supplemented with 100 μg/ml kanamycin, followed by inoculation at a dilution of 1:100 in 100 ml fresh LB medium containing kanamycin and aerobic incubation at 37°C. When OD_600_ reached 0.6, isopropyl -β-D-1-thiogalactopyranoside (IPTG) was added to the growth medium at a final concentration of 0.5 mM. After further incubation overnight at 16°C, the cells were harvested by centrifugation at 8,000×g for 10 min at 4°C.

### Purification and identification of the recombinant LipG1

The lipase was purified with protocol described in detail previously [19]. The bacterial pellets were resuspended in 15 ml lysis buffer (50 mM Tris-Cl pH 8.0), disrupted by sonication, followed by centrifugation at 10,000 × g for 30 min at 4°C. The concentrated supernatant (crude enzyme) was applied to the Ni-NTA affinity chromatography column (GE Healthcare Life Sciences, Piscataway, USA) which was equilibrated with washing buffer (50mMTris-ClpH8.0, 500 mM NaCl, and 10% glycerol). The enzyme protein was eluted using an imidazole gradient (0, 20, 40, 80, 100, and 200 mM) in washing buffer. After the elution, the imidazoles in the purified enzyme were removed by dialysis in 50 Mm Tris-Cl (pH 8.0). The purified enzyme was analyzed by SDS-PAGE. The single protein band after purification was confirmed by the peptide mass fingerprinting.

### Lipase activity assay

Lipase activity was determined using the chromometer method described by Zhang et al. [19]. Unless otherwise described, lipase activity was measured at 40°C with 500 μM *p*NP decanoate in 50 mM Tris-HCl buffer (pH 9.0) containing 1% ethanol. The reaction can begin by the addition of 100 μl of purified enzyme at a concentration of 0.25 mg/ml. The reaction mixture was incubated at 40°C for 10 min, after which the reaction was terminated through addition of 1ml ethanol. Blank reactions were performed in reaction mixtures without the enzyme. The mixtures were centrifuged, and the absorbance of the supernatants was measured at 405 nm with spectrophotometer (Beckman, USA). One unit (U) of enzyme activity was defined as the amount of enzyme that releases 1 μ mol of *p*NP per minute.

### Enzyme characterization of recombinant LipG1

The *p*NP decanoate was used to assay the following characteristics of LipG1. The optimal pH was determined at 40°C using buffers with pH from 3.0 to 12.0, i.e., 2 0 mM disodium hydrogen phosphate-citric acid buffer (pH 3.0–8.0), 50 mM Tris—HCl buffer (pH 8.0–10.0), and 20 mM glycine-NaOH (pH 10.0–12.0). To test its pH stability, 100 μl of the lipase (2.5 mg/ml) was preincubated for 1 h at 40°C in 900 μl buffers with pH values ranging from 3.0 to 12.0, and then lipase activity was measured under standard conditions. The enzyme’s optimal temperature was determined by measuring its activity at temperatures ranging from 0°C to 60°C, with pH set as 8.0. The thermostability was monitored by preincubation of the enzyme in Tris-HCl buffer (pH 8.0) for 30, 60, 90, and 120 min at 20, 30, 40, and 50°C. Lipase activity was then measured under standard conditions. The effects of different metal ions, chemical reagents, detergents, and organic solvents on enzymatic activity were assessed in 50 mM Tri-HCl buffer (pH 8.0) at 40°C. The reactions contained 1 or 10 mM of NaCl, KCl, BaCl_2_, CaCl_2_, CoCl_2_, CuCl_2_, NiCl_2_, MgCl_2_, MnCl_2_, ZnCl_2_, CdCl_2_; 0.1 or1% Tween-20, Tween-40, Tween-80, Triton X-100, SDS, and cetyltrime-thylammonium bromide (CTAB); 10% or30% methanol, ethanol, isopropanol, capryl alcohol, n-heptane, glycerol and dimethyl sulphoxide (DMSO).

In order to examine the resistance to different proteases, the purified recombinant enzyme (100 μg ml^−1^) was incubated with 10 μg ml^−1^ trypsin (from bovine, pH 7.6, 25°C, 14,700 U mg^−1^; Sigma), 250 μg ml^−1^ α-chymotrypsin (type II from bovine, pH 7.8, 25°C, ≥40 U mg^−1^; Sigma), 500 μg ml ^−1^subtilisin A (type VIII from *Bacillus licheniformis*, pH 7.5, 37°C, 10 U mg^−1^; Sigma), 330 μg ml ^−1^proteinase K (type VIII from *B*. *licheniformis*, pH 7.5, 37°C, 30 U mg ^−1^; Amresco, Solon, USA), respectively. After incubation for 1 or 2 h, the residual activity was measured under standard assay conditions. For the control sample, the recombinant enzyme was incubated under the same conditions without protease.

The substrate selectivity of LipG1 for *p*NP esters was assayed using *p*NP acetate (C2), *p*NP butyrate (C4), *p*NP octanoate (C8), *p*NP decanoate (C10), *p*NP laurate (C12), *p*NP myristate (C14), and *p*NP palmitate(C16) as substrates in 50 mM Tris-HCl (pH 8.0) at 40°C. The *p*NP ester substrates except *p*NP palmitate were dissolved in ethanol at a final concentration of 50 mM as the stock solution, and *p*NP palmitate was dissolved in isopropanol. The working concentration range of the *p*NP esters was from 0.01 to 2 mM. Initial velocity versus substrate concentration data were fitted to the Lineweaver—Burk transformation of the Michaelis—Menten equation.

### Experimental diets and feeding experiment

Five isonitrogenous and isocaloric experimental diets were formulated containing approximately 34% crude protein, 9% crude lipid (S1 Table).Diet with palm oil as the lipid source was used as negative control (CKn), while soybean oil diet was positive control group (CKp). Palm oil diet were supplemented with 3 U g^-1^ LipG1, 6 U g^-1^ LipG1 and 6 U g^-1^commercial lipase (Leaveking, Shengzhen, China), giving treatment groups T1, T2 and M, respectively. The feed pellets were dried under forced air at room temperature for 24 h and then kept at 4°C.

Common carp (*Cyprinus carpio*) were obtained from Tianjing freshwater fish husbandry factory (Tianjing, China). Before starting the experiment, the juvenile common carp were reared in an experimental thermo regulated rearing system and fed with the control diet for 2 weeks to acclimatize to the experimental conditions. Fish of similar sizes (5.66 ± 0.21 g) were randomly distributed into 15 circular glass tanks (30 l) and each glass tank was stocked with 12 fish. Each diet was then randomly assigned to triplicate tanks. The fish were hand-fed to apparent satiation three times daily (at 8:30, 12:30 and 16:30) for 28 days. During the experimental period, the water temperature, dissolved O_2_, pH and ammonia content were maintained at 28.0 ± 1.3°C, 7.60 ± 0.28 mg l^−1^, 7.5 ± 0.29 and 0.10 ± 0.02 mg l^−1^, respectively.

### Sampling and measurements

At the end of the feeding experiment, all of the fish were anesthetized with MS-222. Body weight was measured at batch. The hepatopancreas and gut were rapidly excised from three fish of each tank. The samples of fish from the same tank were pooled, frozen in liquid nitrogen and stored at −80°C. Weight gain (WG), feed conversion ratio (FCR) and survival rate (SR) were calculated.

### Lipid-metabolism-related enzymes activities

The hepatopancreas and gut samples were homogenized in 4 volumes of ice-cold buffer (20 mM Tris-HCl, 0.25 M sucrose, 2 mM EDTA, pH 7.4) and centrifuged at 18,000 × g for 10 min at 4°C. Lipase and protease activities were measured in the homogenate with commercial assay kits (Jiancheng Biotech. Co., Nanjing, China).

### Adhesive intestinal bacterial community

The DNA was extracted from pooled gut samples in each treatment using the method according to He et al. [23]. The PCR-DGGE method was according to Zhou et al. [24]. The V3 region of the 16S rRNA gene was amplified with the primers 338-GC-f and 519r. DGGE was performed using a D-Code universal Mutation System (BioRad). Purified PCR products were loaded onto denaturing gradients ranging from 40 to 60%, and then electrophoresis was conducted with a constant voltage of 65 V for 15 h in 1×TAE buffer at 60°C. The gels were stained in an ethidium bromide solution (0.5μg/mL in TAE buffer) and destained in distilled water for 20 min, respectively, and viewed by UV transillumination. The excised bands were reamplified and purified, then sequenced.

### Statistics

The effects of different diets in the feeding experiment were analyzed with one-way ANOVA, and Duncan’s multiple range test was used to compare the means between any two groups. Differences with a *P* value lower than 0.05 were considered as significant. All the statistical analysis was conducted on SPSS 17.0.

### NCBI accession numbers

The nucleotide sequence of the chitinase gene (*lipG1*) was deposited in GenBank under the accession number KM925083.

## Results

### Sequence analysis of LipG1

**Fig 1:**
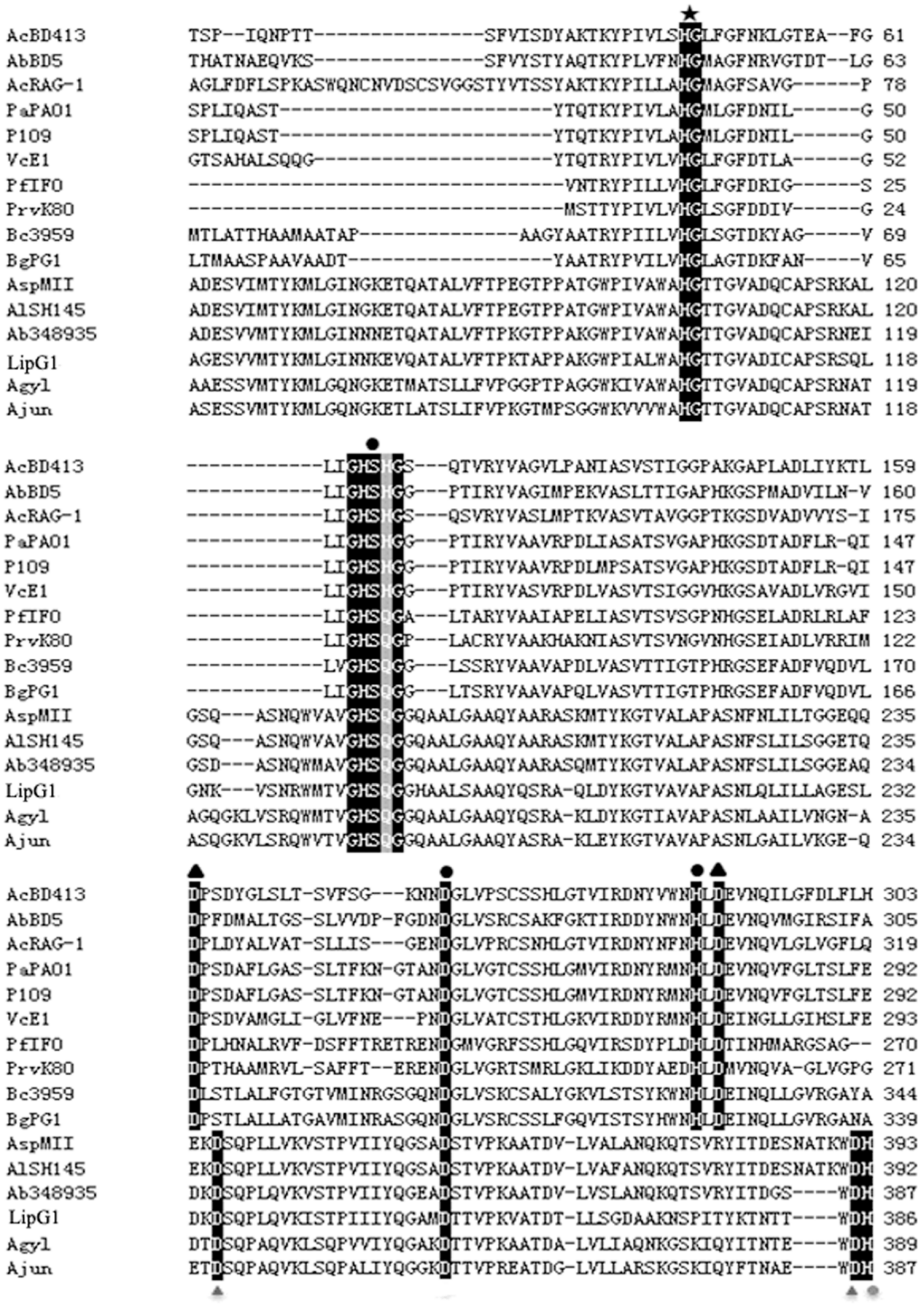
Alignment of amino acid sequences of representative lipases from subfamilies I.1 and I.2 of bacterial lipases and the group of lipases represented by LipG1. Abbreviations and sequence accession numbers: AcBD413 (*Acinetobacter calcoaceticus* BD413), CAA56780.1; AbBD5 (*A*. *baumannii* BD5), ABW70205.1; AcRAG-1 (*A*. *calcoaceticus* RAG-1), AAD29441.1; PaPAO1 (*Ps*. *aeruginosa* PAO1), CAA44997.1; P109 (*Pseudomonas* sp. 109), P26877.1; VcE1 (*V*. *cholerae* E1), P15493.2; PfIFO (*P*. *fragi* IFO-12049), P08658.2; PrvK80 (*Proteus vulgaris* K80), AAB01071.1; Bc3959 (*Burkholderia cepacia* DSM3959), P22088.2; BgPG1 (*Burkholderia glumae* PG1), Q05489.1; AspMII (*Acinetobacter* sp. MII), KGH48978; AlSH145 (*A*. *lwoffii* SH145), WP_004279025; Ab348935 (*A*. *baumannii* 348935), WP_034704719.1; LipG1, this study; Agyl (*A*. *gyllenbergii)*, WP_032860322.1; Ajun (*A*. *junii)*, WP_005402474.1. Symbols: ● Catalytic triad residues, ▲ Asp residues involved in calcium binding, ★ HG dipeptide of the oxyanion loop, ○ Putative triad residue, △Putative calcium binding Asp residues.

Bioinfomatics analysis showed that the *lipG1* gene (KM925083) contained an open reading frame with 1,227 nucleotides, encoding a protein of 406 amino acids (Fig A in S2 File). A 20 amino acid residue signal peptide was found at the N-terminal of the lipase with a cleavage position between Cys20 and His21 as predicted by Signal P (Fig A in S2 File). BLASTp analysis indicated that LipG1 was 99% identical to hypothetical protein from *Acinetobacter tandoii* (WP_016167430.1), and was 70%, 68%, 68%, 62% and 61% identical to lipases from *A*. *baumannii* 348935 (WP_034704719.1), *Acinetobacter* sp. MII (KGH48978.1), *A*. *lwoffii* SH145(WP_004279025), *A*. *gyllenbergii* (WP_032860322.1) and *A*. *junii* (WP_005402474.1), respectively. Alignment of LipG1 and its relatives with representatives of subfamily I.1 and I.2 of bacterial true lipases revealed the typical lipase semi-conserved pentapeptide, GXSXG, where the catalytic residue Ser is located, as well as the catalytic residue Asp, at homologous positions in all sequences (Fig 1). The last catalytic residue constituting the lipase consensus catalytic triad, His, was not found at homologous position in LipG1. Nevertheless, a conserved His residue was located in LipG1 and its relatives at the position close to the catalytic His for I.1 and I.2 lipases (Fig 1). Therefore, the consensus lipase catalytic triad in LipG1putatively comprises of Ser 190 (in the motif GHSQG), Asp 354 and His 386, which requires further experimental confirmation. An HG sequence, which putatively constitutes an oxyanion hole in the three-dimensional protein structure, was also located at homologous position for all sequences (Fig 1).

### Enzyme expression, purification, and mass spectrometry analysis

The recombinant LipG1 was expressed in *E*. *coli* BL21 in the presence of 0.01mM IPTG at 20°C. After Ni-NTA affinity chromatography, the enzymatic activity of recombinant LipG1 was 8908 U mg^−1^ toward C8 *p*NP. The purified enzyme presented as a single band of about 40kDa on SDS-PAGE (Fig B in S2 File), which matched the expected molecular weight. The band was excised from the SDS-PAGE, digested with trypsin, and analyzed by LC—ESI—MS/MS for peptide finger printing. The amino acid sequences obtained from the mass peaks were compared with LipG1. The peptides WMTVGHSQGGHAALSAA, TFTALITAGLRNPNPSLQYSQVFK and TQSNFMTLSPVKKFLDTDSQPLQVKISTPIIIYQ completely matched the sequences of corresponding LipG1 fragments, confirming that the purified protein was recombinant LipG1 (Fig C in S2 File).

### Effects of pH and temperature on LipG1 activity

**Fig 2:**
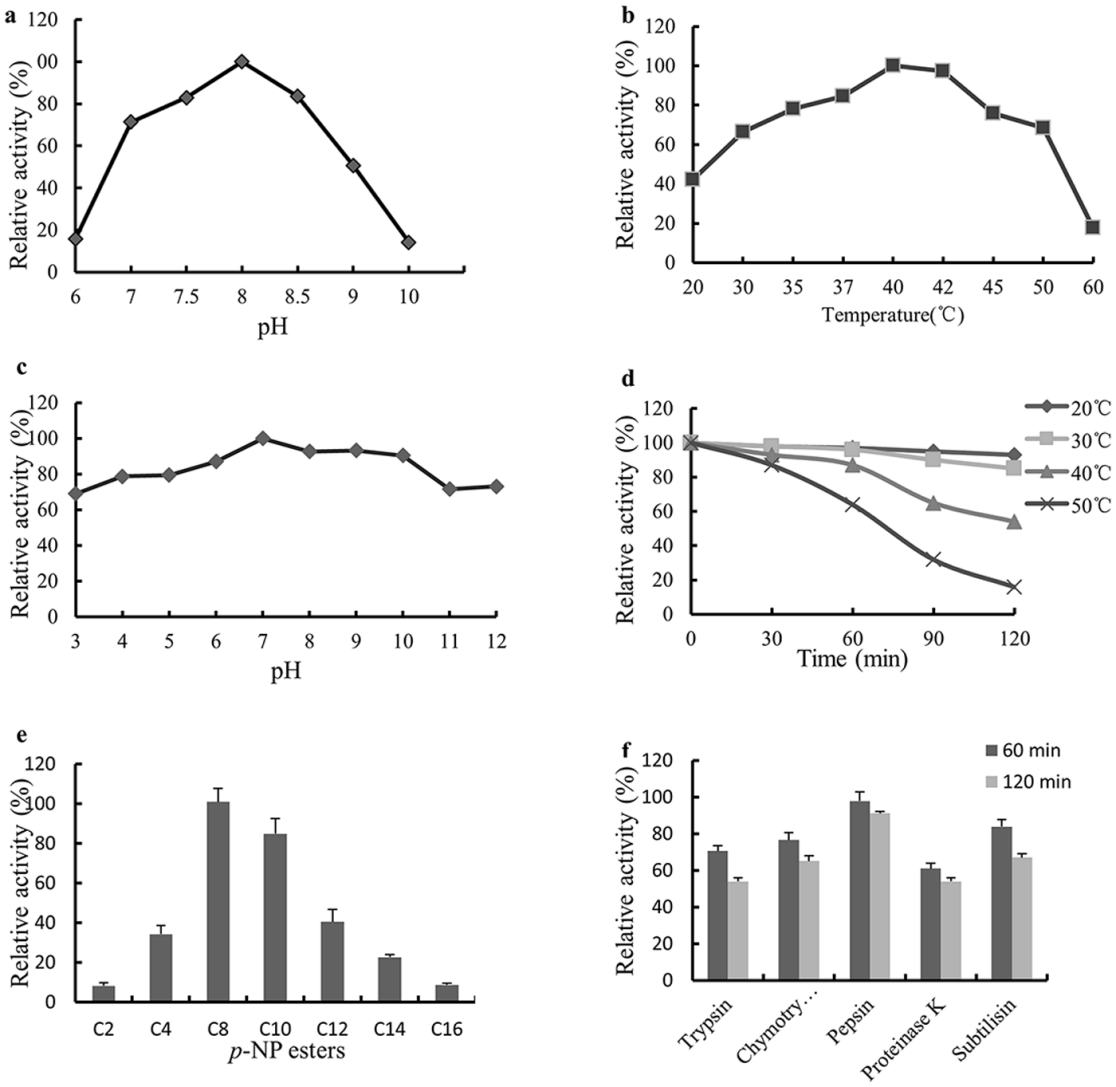
Characterization of purified recombinant LipG1. a. The effect of pH on lipase activity. The activity assay was performed at 37°C in buffers of pH 3.0–12.0 for 10 min. b. The effect of temperature on lipase activity measured in 0.1 M PBS buffer (pH 8.0) for 10 min. c. pH stability of LipG1. After pre-incubating the enzyme at 37°C for 1 h at various pHs, the residual activity was measured in 0.1 M PBS buffer (pH 8.0), 40°C. d. Thermostability of purified LipG1. The enzyme was preincubated at 20, 30, 40, or 50°C in 0.1 M PBS buffer (pH 8.0). Aliquots were removed at specific time points for measurement of residual activity in the same buffer at 40°C. e. Substrate specificity of LipG1 determined with various chain length fatty acid esters. f. Effects of proteases on the activity of LipG1. Each value in the panel represents the mean ± SD (n = 3).

LipG1showed maximum activity at pH 8.0, and retained at least 70% of its maximum activity between pH 7.0 and 8.5 (Fig 2a). LipG1 was stable over a wide pH range, remaining at least 60% of its maximum activity after incubation at pH from 3.0 to 12.0 (Fig 2c). The enzyme activity of LipG1 peaked at 40°C, and retained over 40% of its maximum activity at temperatures from 20°C to 35°C (Fig 2b). The recombinant lipase showed high stability at 20°C and 30°C. However, the activity decreased quickly when incubated at 50°C (Fig 2d).

### Effects of metal ions, organic solvents, detergents and proteases on LipG1 activity

The effects of different metal ions, organic solvents, and detergents on the activity of LipG1 are shown in Table 2, Tables A and B in S1 File, respectively. At the concentration of 1mM, lipase activity was strongly inhibited byCo^2+^, Cr^3+^, Fe^3+^,Pb^+^, Ag^+^ and EDTA, and enhanced by Ca^2+^, Mg^2+^. At the higher concentration of 10 mM, Cu^2+^, EDTA,Co^2+^, Zn^2+^ and Mn^2+^ drastically inhibited its activity; Na^+^, K^+^, Cr^3+^, Ni^2+^, Pb^+^ and Ag^+^ strongly inhibited the activity. Nevertheless, a stimulating effect was still observed for Ca^2+^ and Mg^2+^ (Table 2). The enzyme was stable in various organic solvents at the concentration of 10%, with extended stability at the concentration of 30% for methanol, ethanol, n-heptane, glycerol and DMSO (>75% activity remained). However, isopropanol and capryl alcohol drastically reduced its activity at the concentration of 30% (Table A in S1 File). At the concentration of 0.1%, Tween 80 and CTAB didn’t significantly inhibit the activity of LipG1, and the non-ionic detergent Triton X-100 slightly enhanced its activity. Tween 20, Tween 40, SDS reduced its activity by 37.5%, 28.3%, 57.7%, respectively. At the concentration of 1%, all the detergents except Triton X-100 drastically inhibited the lipase activity, with over 50% activity loss observed (Table B in S1 File).

**Table 2:** Effects of various metal ions on LipG1 activity.

Metal ion	Relative activity (%)	
	1 mM	10 mM
Na^+^	101.6±2.3	88.7±1.7
K^+^	107.4±1.9	81.6±2.3
Ca^2+^	115.5±±4.4	213.9±3.5
Zn^2+^	97.5±±2.1	37.7±2.1
Li^+^	104.2±2.5	97.5±2.5
Co^2+^	50.6±1.2	30.3±0.9
Cr^3+^	79.6±2.2	70.4±1.7
Ni^2+^	81.1±3.1	49.2±1.2
Cu^2+^	84.9±±2.2	0
Mg^2+^	119.4±2.7	171.0±1.4
Fe^3+^	66.9±1.8	18.2±0.6
Mn^2+^	93.7±2.0	15.1±0.9
Pb^+^	76.6±1.5	56.8±1.3
Ag^+^	60.6±1.2	43.4±1.5
EDTA	75.5±1.3	11.1±0.4

We also investigated the resistance of recombinant LipG1against proteases (Fig 2f). The enzyme retained over 60% activity after treatment with all proteases at 37°C for 1 h, with over 80% activity retained for subtilisin and pepsin. Also, longer incubation time of 120 min didn’t lead to considerable further activity loss for all the tested proteases.

### Substrate specificity of LipG1

The substrate specificity of LipG1 was examined using *p*NP esters with different chain lengths. Results showed that the lipase showed activity on a wide range of substrates. LipG1 preferred medium chain fatty acid substrates, and the highest activity was registered for *p*NP octanoate (Fig 2e, S2 Table).

### Effect of LipG1 on growth and feed utilization of common carp

Dietary supplementation of LipG1 at 6 U/g significantly increased the final body weight (FBW) and weight gain (WG) of common carp compared with negative control after feeding for 4 weeks. LipG1 at 3 U/g and commercial lipase at 6 U/g marginally increased the growth of carp compared with the negative control. Overall, all the lipase supplementation groups showed similar growth performance (*P* > 0.05), which is comparable to the group with soybean oil (Table 3). Also, significantly lower FCR (feed conversion ratio) was observed in 6 U/g LipG1 group compared with the negative control (*P* < 0.05), and marginally decreased FCR was recorded in 3 U/g LipG1 and the commercial lipase groups, with no significant difference among the lipase addition groups, which were comparable to the soybean oil group(Table 3).

**Table 3:** Effects of dietary lipase on weight gain, feed conversion ratio and survival rate of common carp (*Cyprinus carpio*) (n = 3).

	CKn	T1	T2	M	CKp
IBW (g)	2.09±0.04^a^	2.14±0.02^a^	2.16±0.01^a^	2.13±0.01^a^	2.14±0.02^a^
FBW (g)	4.12±0.12^a^	4.4±0.05^ab^	4.58±0.12^b^	4.44±0.08^ab^	4.45±0.06^ab^
WG (%)	96.7±2.17^a^	109.18±3.90^ab^	112.32±4.67^b^	108.4±3.58^ab^	108.25±1.37^ab^
FCR	2.03±0.05^a^	1.80±0.06^ab^	1.75±0.07^b^	1.81±0.06^ab^	1.81±0.02^ab^
SR (%)	100	100	100	100	100

Values in the same row with different superscript letters (a, b, ab) have significant difference (*P*<0.05). FBW, final body weight (g); Weight gain (%), WG (%) = 100 × (Final body weight−Initial body weight)/Initial body weight; Feed conversion ratio, FCR = feed fed (g)/weight gained (g).

### Effect of LipG1 on digestive enzyme activities of common carp

Heptaopancreas trypsin activity was not significantly different among groups, while the gut trypsin activity was similar among the palm oil groups regardless of lipase supplementation, and the activity in soybean oil group was significantly higher (Table 4). Dietary exogenous lipases (T1, T2 and M) significantly increased gut lipase activity compared with the negative control (*P* < 0.05), with an activity comparable to the positive control. The heptaopancreas lipase activity was significantly increased in groups with lipase supplementation at 6 U/g (T2 and M) (Table 4). For both gut and heptaopancreas lipase activity, the maximal value was observed in the 6 U/g LipG1 group.

**Table 4:** Trypsin and lipase activities in the gut and heptaopancreas of common carp (*Cyprinus carpio*) fed with different diets (n = 3).

Treatments	CKn	T1	T2	M	CKp
Trypsin activity (U/mg)					
Heptaopancreas	127.7±2.4^a^	125.3±,3.8^a^	128.7±4.3^a^	125.3±2.3^a^	134.3±1.5^a^
Gut	77.0±1.5^a^	78.0±2.3^a^	79.7±3.0^a^	79.3±2.4^a^	86.7±1.5^b^
Lipase activity (U/g)					
Heptaopancreas	62.0±0.6^a^	65.7±1.2^ab^	68.7±2.9^b^	68.7±2.0^b^	67.3±2.0^ab^
Gut	41.3±2.0^a^	49.7±1.8^b^	58.3±1.5^c^	54.0±1.7^bc^	54.0±1.7^bc^

Values in the same row with common superscript (a, b, ab, c, bc) are not significantly different (*P* > 0.05).

### Effect of LipG1 on common carp gut microbiota

**Fig 3:**
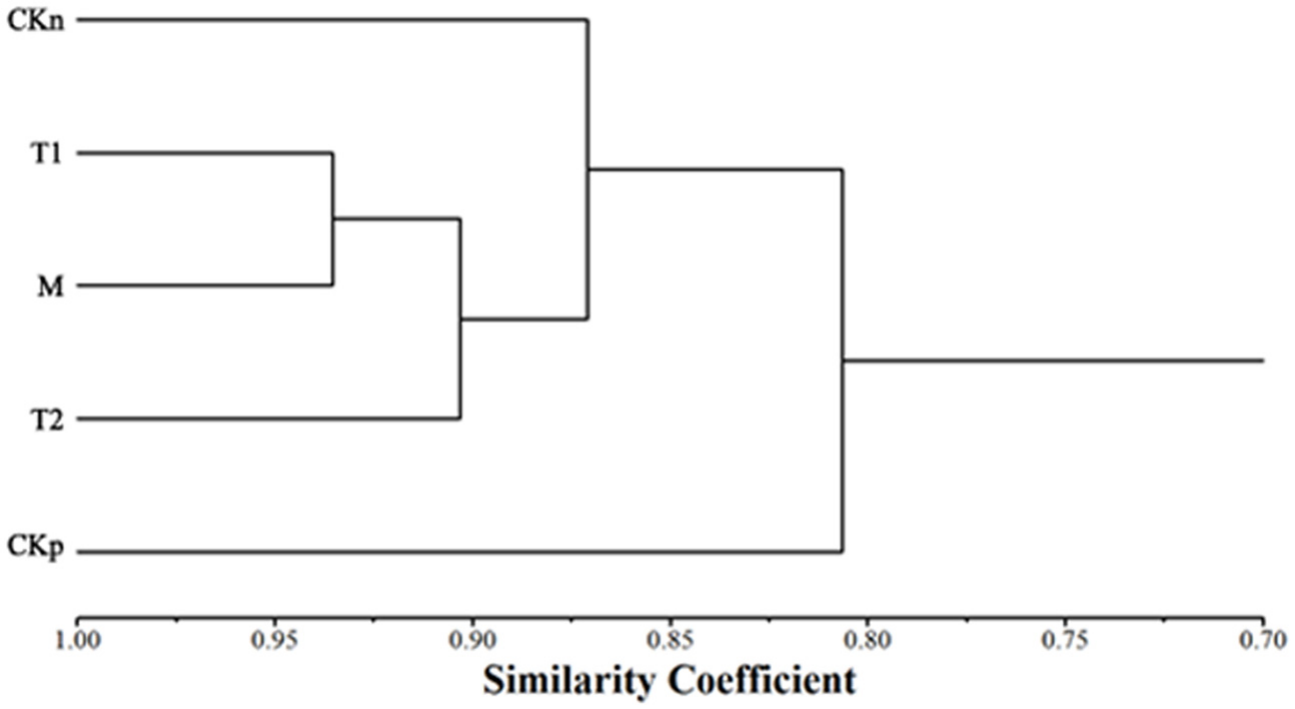
Cluster analysis of the adhesive gut bacterial communities of common carp (*Cyprinus carpio*).

The 16S rRNA gene V3 region PCR-DGGE fingerprints of the adhesive intestinal microbiota of different groups are shown in S1 Fig Most of the OTUs of the intestinal microbiota of common carp were assigned to the *Proteobacteria* (17 OTU) and *Firmicutes* (4 OTU) (S3 Table). The gut microbiota was affected by dietary lipid sources, as the microbiota of palm oil diet associated groups were clearly distinguishable from the microbiota of the soybean oil group (Fig 3). Lipase supplementation didn’t exert significant change to the intestinal microbiota, and the intestinal microbial profiles of all the lipase supplement groups were similar (Fig 3).

## Discussion

**Fig 4:**
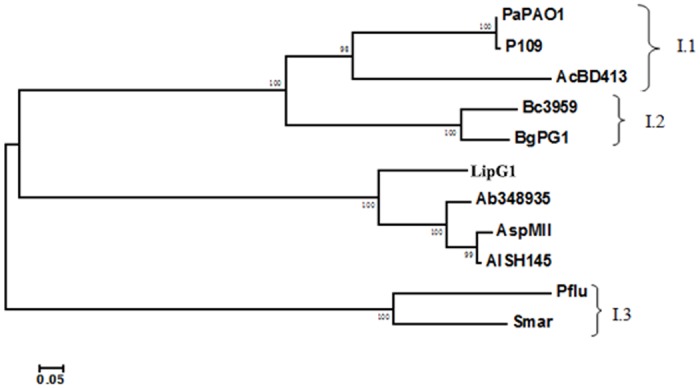
Phylogenetic tree of true lipases produced by Gram-negative bacteria, including LipG1 and its relatives. Abbreviations and sequence accession numbers: PaPAO1 (*P*. *aeruginosa* PAO1), CAA44997.1; P109 (*Pseudomonas* sp. 109), P26877.1; AcBD413 (*Acinetobacter calcoaceticus* BD413), CAA56780.1; Bc3959 (*Burkholderia cepacia* DSM3959), P22088.2; BgPG1 (*Burkholderia glumae* PG1), Q05489.1; LipG1, this study; Ab348935 (*A*. *baumannii* 348935), WP_034704719.1; AspMII (*Acinetobacter* sp. MII), KGH48978; AlSH145 (*A*. *lwoffii* SH145), WP_004279025; Pflu (*P*. *fluorescens* SIK W1), BAA02012.1; Smar (*Serratia marcescens*), BAA02519.1.

Bacterial true lipases comprise the family I of bacterial lipolyitc enzymes, and were further classified into six subfamilies [25]. True lipases from Gram-negative bacteria were assigned into the first three subfamilies. Subfamily I.1 were lipases from *Pseudomonas aeruginosa*, *Vibrio cholerae*, *Acinetobacter calcoaceticus*, *etc*. Subfamily I.2 mainly comprises lipases produced by *Burkholderia*, while I.3 contains enzymes from two distinct species: *Ps*. *fluorescens* and *Serratia marcescens* [25]. Many lipase genes have been cloned from *Acinetobacter* [14, 15, 17, 18, 19]. The *Acinetobacter* lipases were assigned to subfamily I.1 of bacterial true lipases [25], and were proposed as a separate ‘*Acinetobacter*’ clade based on similarity of amino acid sequences [15, 26]. However, amino acid sequence alignment showed that LipG1 isn’t homologous to any of the above mentioned *Acinetobacter* lipases and doesn’t belong to the typical ‘*Acinotebacter*’ lipase clade. Based on the phylogenetic tree predicted from multiple sequence alignment of the lipases (Fig 4), and considering the deviation in the position of the catalytic residue His (Fig 1), we propose that LipG1 and its relatives comprise another subfamily of true lipases from Gram-negative bacteria. The lipases homologous with LipG1 in the BLASTp list all originated from genomic sequence analysis rather than experimental characterization. Also, around half of the hits with over 60% identity in the BLASTp list were designated as hypothetical proteins from *Acinetobacter* strains. Therefore, LipG1 represents a novel group of lipases that deserves further characterization.

LipG1 is most active at 40°C, and retained over 40% of its optimal activity at temperatures from 20°C to 35°C, the temperature range for warm-water aquaculture. Activity of LipG1 peaked at pH 8.0, and showed strong activity in a narrow alkaline pH range, which is similar with other *Acinetobacter* lipases [14, 16, 17]. Remarkably, LipG1 exhibited stability over a wide range of pH from 3.0–12.0. In contrast, incubation of many lipases at acidic pH lower than 5.6 resulted in inactivation [16, 26]. The stability of LipG1 at acidic pH is a useful characteristic as feed additive, as the enzyme has to pass through the acidic gastric environment of the host. In line with other lipases from *Acinetobacter*, the activity of LipG1 was activated by Ca^2+^ and Mg^2+^, while inhibited by Zn^2+^ and Cu^2+^ [16, 18, 19, 27]. Particularly, the positive effect of Ca^2+^ on enzyme stabilization and activity was common in *Acinetobacter* lipases, most probably due to a function of the Ca^2+^-binding pocket, leading to correct active-site configuration [26]. The Ca^2+^-binding Asp residues were conserved in subfamily I.1 and I.2 lipases [15, 25]. Alignment didn’t show putative Ca^2+^-binding Asp residues at the conserved positions in LipG1 (Fig 1). However, in both regions, an Asp residue was located in the close position, which probably constitutes the Ca^2+^-binding pocket (Fig 1). Another notable characteristic of LipG1 is the strong resistance to proteases, which is rarely reported for *Acinetobacter* lipases [28]. Collectively, the ion, protease resistance and strong pH stability of LipG1 supported it as a candidate aquafeed additive enzyme. Moreover, the stability of lipase in the presence of surfactants and organic solvent suggests its potential application in industry.

The overall lipid digestibility may be reduced by high saturates diets [3], such as the diet with palm oil as the sole lipid source. The lowered lipid digestibility may compromise the growth performance of fish, as indicated in the data of our study, where the weight gain and feed efficiency were both marginally reduced in the group with palm oil as the lipid source, compared with the soybean oil group. The feeding experiment data in this study showed that supplementation of exogenous lipase efficiently improved the heptaopancreas and gut lipase activity of common carp. The increased lipolytic activity led to improved feed efficiency and growth performance of the palm oil group of fish, which were comparable to the group with soybean oil as the lipid source. Moreover, lipase supplementation didn’t exert significant change to the intestinal microbiota, indicating that it doesn’t have negative effects on the intestinal health status as reflected by the microbiota. Although several *Acinetobacter* lipase genes were cloned from the intestinal DNA sample of common carp, *Acinetobacter* sp. was recovered only in T1 group in the DGGE fingerprint, suggesting the variation of its occurrence in the microbiota of common carp. Also, the phenomenon may be attributed to the limitation of DGGE in describing the microbiota of ecological samples, especially for low abundance species. Study on the effects of exogenous lipase on lipid digestion and growth is rare in aquatic animals. Koven reported that the supplementation of lipase increased the incorporation of oleic acid in tissues of gilthead seabream larvae [29]. In adult rainbow trout, no effect on growth performance and related parameters were registered with lipase addition [11]. In both research, traditional lipid source was used, and the issue of high dietary palm oil supplementation was not addressed. To our knowledge, this is the first study investigating the effect of supplementation of exogenous lipase on the lipid digestibility and performance of fish fed diet with high level of palm oil. The working temperature range, stability at broad pH range, and resistance to metal ions and proteases of LipG1 make it a suitable exogenous lipase as aquafeed additive. Study is underway to further improve the lipolytic efficiency of LipG1 on palm oil.

In this study, the lipase LipG1 exhibited efficient lipolytic activity at environmental factors generally encountered in the warm water aquaculture, and resistance against acidic pH, metal ions and proteases. Amino acid sequence analysis showed that LipG1 and its relatives represent a novel group of uncharacterized lipases. Supplementation of LipG1significantly improved the gut lipase activity, feed conversion ratio, and growth performance of common carp fed diet with high amount of palm oil, to a level comparable to the group of fish fed with soybean-oil-based diet. Collectively, these results paved the way for further application of LipG1 as aquafeed additive enzyme.

## Supporting Information

S1 Fig Fingerprints of 16S rRNA gene V3 DGGE of the adhesive gut bacterial communities in common carp (*Cyprinus carpio*). (DOCX)

S1 File Effects of various organic solvents (Table A) and detergents (Table B) on LipG1 activity. (DOCX)

S2 File Characterization of LipG1. **(Fig A):** The nucleotide sequence and deduced amino acid sequence of *lipG1*. The amino acid sequence of LipG1 is below the nucleotide sequence. The putative signal peptide sequence is underlined. The stop codon is marked by an asterisk. **(Fig B):** SDS-PAGE of purified LipG1. M, protein molecular mass markers (kDa); lane 1, purified LipG1 protein. **(Fig C):** Peptide mass fingerprint generated by MALDI-TOF mass spectrometry of the products produced by trypsinisation of the LipG1.
(DOCX)

S1 Table The basal diet formulation and its calculated chemical compositions. (DOCX)

S2 Table Kinetic parameters for LipG1. (DOCX)

S3 Table Representative of intestinal adhesive bacteria from common carp (*Cyprinus carpio*). (DOCX)
