# Supplementary material for: A Novel Lipase as Aquafeed Additive for Warm-Water Aquaculture
Source: PLoS One. 2015 Jul 6;10(7):e0132049. doi: 10.1371/journal.pone.0132049 (PMC4492967; doi:10.1371/journal.pone.0132049)
Supplement: S1 Fig — (DOCX) [file pone.0132049.s001.docx]

**S1** **Fig. Fingerprints of 16S rRNA gene V3 DGGE of the adhesive gut bacterial communities in common carp (*Cyprinus carpio*).**
