# Supplementary material for: A Novel Lipase as Aquafeed Additive for Warm-Water Aquaculture
Source: PLoS One. 2015 Jul 6;10(7):e0132049. doi: 10.1371/journal.pone.0132049 (PMC4492967; doi:10.1371/journal.pone.0132049)
Supplement: S1 File — (DOCX) [file pone.0132049.s002.docx]

A．

| Organic solvents | Relative activity (%) | |
| --- | --- | --- |
|  | 10% v/v | 30% v/v |
| Methanol | 109.5±2.8 | 78.5±2.4 |
| Ethanol | 98.2±3.2 | 101.7±2.9 |
| Isopropanol | 100.3±2.4 | 33.8±1.1 |
| Capryl alcohol | 88.1±2.2 | 41.3±1.5 |
| n-heptane | 109.7±3.0 | 110.9±2.6 |
| Glycerol | 93.9±1.8 | 75.5±2.3 |
| DMSO | 114.3±3.1 | 108.3±2.6 |

B．

| Detergents | Relative activity (%) | |
| --- | --- | --- |
|  | 0.1% v/v | 1% v/v |
| Tween20 | 62.5 ± 1.4 | 22.2 ± 1.2 |
| Tween40 | 71.7 ± 1.5 | 33.6 ± 1.0 |
| Tween80 | 94.2 ± 2.4 | 45.1 ± 1.5 |
| Triton X-100 | 121.0 ± 2.6 | 99.0 ± 1.9 |
| SDS | 42.3 ± 1.6 | 0 |
| CTAB | 84.3 ± 1.5 | 31.6 ± 1.2 |

**S1 File. Effects of various organic solvents (Table A) and detergents (Table B) on LipG1 activity.**
