# Supplementary material for: A Novel Lipase as Aquafeed Additive for Warm-Water Aquaculture
Source: PLoS One. 2015 Jul 6;10(7):e0132049. doi: 10.1371/journal.pone.0132049 (PMC4492967; doi:10.1371/journal.pone.0132049)
Supplement: S2 File — (Fig A): The nucleotide sequence and deduced amino acid sequence of lipG1. The amino acid sequence of LipG1 is below the nucleotide sequence. The putative signal peptide sequence is underlined. The stop codon is marked by an asterisk. (Fig B): SDS-PAGE of purified LipG1. M, protein molecular mass markers (kDa); lane 1, purified LipG1 protein. (Fig C): Peptide mass fingerprint generated by MALDI-TOF mass spectrometry of the products produced by trypsinisation of the LipG1. (DOCX) [file pone.0132049.s003.docx]

**A.**


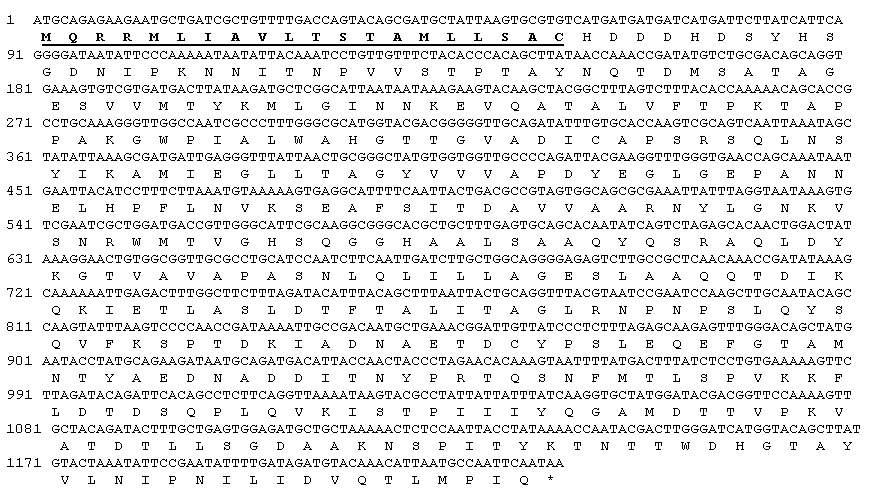


**B.**


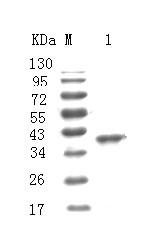


**C.**

1 MQRRMLIAVL TSTAMLLSAC HDDDHDSYHS GDNIPKNNITNPVVSTPTAY

51 NQTDMSATAG ESVVMTYKML GINNKEVQATALVFTPKTAPPAKGWPIALW

101 AHGTTGVADI CAPSRSQLNS YIKAMIEGLL TAGYVVVAPD YEGLGEPANN

151 ELHPFLNVKS EAFSITDAVV AARNYLGNKV SNRWMTVGHS QGGHAALSAA

201 QYQSRAQLDY KGTVAVAPAS NLQLILLAGE SLAAQQTDIK QKIETLASLD

251 TFTALITAGL RNPNPSLQYS QVFKSPTDKI ADNAETDCYP SLEQEFGTAM

301 NTYAEDNADD ITNYPRTQSN FMTLSPVKKF LDTDSQPLQV KISTPIIIYQ

351 GAMDTTVPKV ATDTLLSGDA AKNSPITYKT NTTWDHGTAY VLNIPNILID

401 VQTLMP

**S2** **File. Characterization of LipG1. (Figure A):** The nucleotide sequence and deduced amino acid sequence of *lipG1*. The amino acid sequence of LipG1 is below the nucleotide sequence. The putative signal peptide sequence is underlined. The stop codon is marked by an asterisk. **(Figure B):** SDS-PAGE of purified LipG1. M, protein molecular mass markers (kDa); lane 1, purified LipG1 protein. **(Figure C):** Peptide mass fingerprint generated by MALDI-TOF mass spectrometry of the products produced by trypsinisation of the LipG1.
