# Supplementary material for: A Novel Lipase as Aquafeed Additive for Warm-Water Aquaculture
Source: PLoS One. 2015 Jul 6;10(7):e0132049. doi: 10.1371/journal.pone.0132049 (PMC4492967; doi:10.1371/journal.pone.0132049)
Supplement: S1 Table — (DOCX) [file pone.0132049.s004.docx]

| Ingredinets | Experimental diets(%) | | | | |
| --- | --- | --- | --- | --- | --- |
|  | CKN | CKP | T1 | T2 | M |
| Soybean meal | 36 | 36 | 36 | 36 | 36 |
| Cottonseed meal | 12.14 | 12.14 | 12.14 | 12.14 | 12.14 |
| Corn protein meal | 17.09 | 17.09 | 17.09 | 17.09 | 17.09 |
| Wheat flour | 24 | 24 | 24 | 24 | 24 |
| Monocalcium phosphate | 1.8 | 1.8 | 1.8 | 1.8 | 1.8 |
| Zeolite powder | 0.3 | 0.3 | 0.3 | 0.3 | 0.3 |
| L-lysine·H2SO4 | 0.7 | 0.7 | 0.7 | 0.7 | 0.7 |
| Methionine hydroxy analog-Ca | 0.21 | 0.21 | 0.21 | 0.21 | 0.21 |
| Silicon chloride choline | 0.2 | 0.2 | 0.2 | 0.2 | 0.2 |
| Vitamin C phosphate | 0.1 | 0.1 | 0.1 | 0.1 | 0.1 |
| Vitamin premix | 0.2 | 0.2 | 0.2 | 0.2 | 0.2 |
| Mineral premix | 0.2 | 0.2 | 0.2 | 0.2 | 0.2 |
| Y_2_O_3_ | 0.1 | 0.1 | 0.1 | 0.1 | 0.1 |
| Palm oil | 7.07 |  | 7.07 | 7.07 | 7.07 |
| Soy oil |  | 7.07 |  |  |  |
| Lip G1 |  |  | 3U/g | 6U/g |  |
| Commercial lipase |  |  |  |  | 6U/g |
| Proximate composition (%) | | | | | |
| Crude protein | 33.8 | 34.2 | 33.9 | 34.1 | 34.2 |
| Crude lipid | 9.3 | 8.9 | 8.9 | 9.1 | 9.0 |
| Ash | 5.4 | 5.6 | 5.9 | 5.7 | 5.6 |
| Moisture | 12.4 | 11.6 | 12.9 | 11.3 | 12.6 |

**S1 Table. The basal diet formulation and its calculated chemical compositions.**
