# Supplementary material for: A Novel Lipase as Aquafeed Additive for Warm-Water Aquaculture
Source: PLoS One. 2015 Jul 6;10(7):e0132049. doi: 10.1371/journal.pone.0132049 (PMC4492967; doi:10.1371/journal.pone.0132049)
Supplement: S2 Table — (DOCX) [file pone.0132049.s005.docx]

| Substrate | C8 | C10 | C12 |
| --- | --- | --- | --- |
| *K*_m_ (mM) | 0.058 ±0.002 | 0.095±0.004 | 0.126±0.005 |
| *k_cat_* (s^-1^) | 981.00±32.45 | 873.00±25.67 | 620.00±17.89 |
| *k_cat_*/*K_m_*(s^−1^mM^−1^) | 16913.00±467.85 | 9189.00±284.63 | 4920.00±186.90 |

**S2 Table. Kinetic parameters for LipG1.**
