# Supplementary material for: A Novel Lipase as Aquafeed Additive for Warm-Water Aquaculture
Source: PLoS One. 2015 Jul 6;10(7):e0132049. doi: 10.1371/journal.pone.0132049 (PMC4492967; doi:10.1371/journal.pone.0132049)
Supplement: S3 Table — (DOCX) [file pone.0132049.s006.docx]

| Phylum | Band NO. | BLASTn Top Hit | Similarity | CKn | T1 | T2 | M | CKp |
| --- | --- | --- | --- | --- | --- | --- | --- | --- |
| Proteobacteria | 2 | *Stenotrophomonasmaltophilia* (JX646653.1) | 99 | + | + | + | + | + |
|  | 3 | *Aeromonas* sp. (JX899623.1) | 99 | + | + | + | + | + |
|  | 4 | Uncultured bacterium (JQ409205.1) | 99 | + | + | + | + | + |
|  | 5 | *Pseudomonas aeruginosa* (JX661716.2) | 100 | + | + | + | + | + |
|  | 6 | Uncultured bacterium (FN659422.1) | 99 | + | + | + | + | + |
|  | 9 | *Aeromonas* sp. (JX899623.1) | 100 | + | + | + | + | + |
|  | 11 | *Pseudomonas* sp. (JX971964 ) | 100 | + | + | + | + | + |
|  | 12 | *Magnetospirillumaberrantis* (JQ673402.1) | 99 | + | + | + | + | + |
|  | 13 | *Psychrilyobacter* sp. (JF825448.1) | 99 | + | + | + | + | - |
|  | 14 | *Mucilaginibacter*sp. (JX089330.1) | 100 | - | - | - | - | + |
|  | 15 | *Pseudomonas* sp. (JX971964 ) | 99 | + | - | - | - | - |
|  | 20 | Uncultured *Ochrobactrum* sp. ( KC110985) | 99 | + | + | + | + | + |
|  | 24 | *Raoultella* sp. (JX908966.1) | 100 | + | + | + | + | + |
|  | 25 | *Ferrimonaskyonanensis* (AB681438.1) | 99 | + | + | + | + | + |
|  | 26 | *Dickeya* sp. (JN709491.1) | 98 | - | - | + | - | - |
|  | 28 | *Acinetobacter*sp. (HF536578) | 99 | - | + | - | - | - |
|  | 31 | Uncultured alpha proteobacterium ( JQ580268 ) | 99 | - | - | - | - | + |
| Firmicutes | 7 | *Lachnospira multipara* (FR733699.1) | 99 | + | + | + | + | + |
|  | 17 | *Clostridium lentocellum* (NR_026101 ) | 99 | + | + | + | + | + |
|  | 18 | Uncultured bacterium (JQ410893) | 100 | - | - | - | - | + |
|  | 22 | Uncultured *Clostridium* sp. (KC197346) | 100 | + | + | + | + | + |
| Fusobacteria | 1 | *Cetobacterium* sp. (HM778168.1) | 100 | - | + | + | + | + |
| Cyanobacteria | 19 | Uncultured cyanobacterium (JQ579927 ) | 99 | - | + | - | + | - |
| Uncultured | 8 | Uncultured bacterium (JX013058.1) | 100 | + | + | + | + | + |
|  | 10 | Uncultured bacterium (HQ120674) | 99 | + | + | + | + | + |
|  | 16 | Uncultured bacterium (JQ186536) | 100 | + | + | + | + | + |
|  | 21 | Uncultured bacterium (JX120502) | 100 | + | + | + | + | + |
|  | 23 | Uncultured bacterium ( FJ302271) | 99 | + | + | + | + | + |
|  | 27 | Uncultured bacterium (HQ218870) | 99 | + | + | + | + | + |
|  | 29 | Uncultured bacterium (JN183382) | 100 | + | + | - | - | - |

**S3 Table. Representative of intestinal adhesive bacteria from common carp (*Cyprinus carpio*).**
